# Dietary Patterns of Nurses on Rotational Shifts Are Marked by Redistribution of Energy into the Nightshift

**DOI:** 10.3390/nu12041053

**Published:** 2020-04-10

**Authors:** Alan Flanagan, Elizabeth Lowson, Sara Arber, Bruce A. Griffin, Debra J. Skene

**Affiliations:** 1 Department of Biochemical Sciences, Faculty of Health and Medical Sciences, University of Surrey, Guildford GU2 7XH, UK; d.skene@surrey.ac.uk; 2 Department of Nutritional Sciences, Faculty of Health and Medical Sciences, University of Surrey, Guildford GU2 7XH, UK; b.griffin@surrey.ac.uk; 3 Department of Sociology, Faculty of Arts and Social Sciences, University of Surrey, Guildford GU2 7XH, UK; elizabeth.lowson@hotmail.com (E.L.); s.arber@surrey.ac.uk (S.A.)

**Keywords:** shift work, circadian rhythms, diet, nutrition, circadian misalignment

## Abstract

Nightshift work is associated with adverse health outcomes, which may be related to eating during the biological night, when circadian rhythms and food intake are misaligned. Nurses often undertake nightshift work, and we aimed to investigate patterns of energy distribution and dietary intake across 14 days in 20 UK National Health Service (NHS) nurses working rotational shifts. We hypothesised that the proportion of daily energy consumed during the nightshift would increase over consecutive nights. Primary and secondary outcome measures included intakes of energy and macronutrients. Our results show that nurses consumed the same total daily energy on nightshifts and non-nightshifts, but redistributed energy to the nightshift period in increasing proportions with a significant difference between Night 1 and 2 in the proportion of total daily energy consumed (26.0 ± 15.7% vs. 33.5 ± 20.2%, mean ± SD; *p <* 0.01). This finding indicates that, rather than increasing total energy intake, nurses redistribute energy consumed during nightshifts as a behavioural response to consecutive nightshifts. This finding informs our understanding of how the intake of energy during the biological night can influence adverse health outcomes of nightshift work.

## 1. Introduction

Substantial evidence now exists for the adverse effects of nightshift work in promoting weight gain and cardiometabolic risk [1,2,3,4]. However, while this association may be expected to relate to an excess of energy driving adiposity, evidence suggests that nightshift workers do not consume more total daily energy in comparison to fixed-day workers [5,6]. A meta-analysis of cross-sectional studies showed no overall difference in the total intake of energy between shift workers and day workers [5]. Similarly, a prospective study of nightshift workers from the same investigators also reported no difference in total energy intake or meal frequency between nightshifts and dayshifts/days-off [6]. Another study found no difference in total energy intake between nightshift workers and day-only workers, and observed greater meal frequency in the former group [7]. 

There is evidence to suggest that the observations of adverse metabolic effects of nightshift work may be explained, in part, by discordance between food intake during the biological night and endogenous circadian rhythms, which are entrained to the 24 h light/dark cycle, and optimised for nutrient intake during the daytime [1]. Circadian misalignment occurs when the environmental light/dark cycle, and behavioural cycles of feeding/fasting and sleep/wake, become misaligned with the endogenous circadian timing system, such as occurs in shift work [1]. Nightshift work is also associated with other risk factors. In particular, the deleterious effects of sleep deprivation on global indices of health [8], coupled to the mistimed intake of food during the biological night, is likely to present a significant metabolic challenge in desynchronising nutrient intake from the normally dichotomous, diurnal patterns of feeding and fasting, and the light/dark cycle [1,9,10,11,12,13].

Cumulatively, these data suggest that the increase in cardiometabolic risk from nightshift work reflects the time-of-day consumption of energy intake, driven by alterations in the patterns and timing of energy intake and diet composition. However, despite extensive literature on energy intake during the nightshift period itself, there is little research on patterns and timing of energy intake, and how these patterns are altered over consecutive nightshifts [4]. Here, we aimed to quantify the pattern of energy intake and redistribution during the nightshift phase in a group of UK National Health Service (NHS) nurses working rotating shift patterns, with a minimum of two consecutive nightshifts. Each nightshift was examined separately, with energy intake being expressed as a proportion of total daily energy. We hypothesised that nurses working rotational night shifts would increase the proportion of energy distributed to the night phase over consecutive night shifts.

## 2. Methods

### 2.1. Participants

Participants were recruited from eight NHS Hospital Trusts in Southern England, who provided recruitment material to their staff. Eligibility criteria included female hospital nurses and midwives (hereafter defined as “nurses”) aged 30–55 years, who were engaged in rotating shifts of no less than 29 h per week. All the participants were mothers with at least one child aged 8–18 years. Their work schedules included day shifts and a minimum of two consecutive night shifts of at least 8 h between 20:00 and 08:00 h. Exclusion criteria included use of sleep medications and lack of English fluency. The present study was a secondary analysis of data collected during the course of previous research (October 2006–September 2008) into the impact of nightshift work on nurses and their families, at the University of Surrey [14,15].

Ethical approval for the study was obtained from the Surrey NHS Research Ethics Committee (REC Ref 06/Q1909/17), and the University of Surrey Ethics Committee (EC/2006/60/SOCIO), and research governance obtained from the eight participating NHS Hospital Trusts. Written informed consent was obtained from all participants.

### 2.2. Study Design

The aim of the study was to examine the impact of rotating night work on women, working as qualified NHS nurses, and their families. The study used a mixed methods approach, with endpoint measures of the quality and amount of sleep, mood ratings, and dietary intake, the latter of which was recorded by each participant in a 14-day diet diary. The findings in relation to sleep quality, cortisol and mood ratings have been previously published [14]. Participants were requested to record the food and/or drinks consumed, the time of consumption, the location of consumption, whether the foods/drinks contained caffeine, who prepared the food, and who the food was eaten with. Participants were requested to complete a food diary on the evening of that day’s intake.

### 2.3. Data Analysis

Primary and secondary outcomes were the intake of energy and macronutrients, respectively. An additional secondary outcome was the mean number of eating occasions during the nightshift. To test for differences in energy intake over sequential nightshifts, nurses were separated by number of consecutive nights: nurses who worked three consecutive nights (*n =* 12) were analysed together (as “3-Night Nurses Group”), while nurses who worked two consecutive nights only (*n =* 8) were grouped and analysed together (as “2-Night Nurses Group”). Because highly significant differences were observed in energy and macronutrient intake between the first and second nightshifts, all nurses were also analysed as a combined group (as “All 2-Night Group”) for differences in these variables.

Dietary intake data were analysed using Nutritics v5.09 [16]. The relevant database utilised for diary entries was the 2015 ‘Composition of Foods Integrated Dataset ‘CoFID’, including McCance and Widdowson’s 7th Edition [17]. To account for diary entries failing to specify a portion size (i.e., in cups, grams, or spoon measures, or in readily identifiable pre-packaged portions), the demographic average in grams or millilitres was entered for a given food or drink. Demographic averages were based on the UK National Diet and Nutrition Survey, which provided data on the average portion of a given food consumed by males and females across different age ranges [17]. This approach provided values that were more representative of real intakes, than that based on UK Food Standards Agency recommended portion sizes. Energy intake was separated into four time-bins, accounting for the approximate duration of the nightshift (21:00–07:00 h) in the group of nurses analysed; “morning” (07:01–12:00 h); “afternoon” (12:01–17:00 h); “evening” (17:01–21:00 h) and “nightshift” (21:01–07:00 h). Mean energy intake was calculated for two conditions, ‘nightshift’ and ‘non-nights’, for each time-bin. Total energy for each nightshift was calculated and organised sequentially, from 21:00–21:00 h the following day to account for the entire nightshift period and include food consumed the day after the nightshift. The energy consumed on each nightshift (21:00–07:00 h) was calculated against the total daily energy intake for that day, and expressed as a percentage of total daily energy intake. This value was used to assess changes in percentage of daily energy consumed during the nightshift over sequential nights. Macronutrient intake was calculated as mean energy derived from carbohydrate, fat, and protein, for each nightshift, and additionally expressed as a percentage of total daily carbohydrate, fat, and protein intake, respectively. Eating occasions were defined as any occasion that food or drink was ingested with a minimum energy content of 50 kcal, separated by 15 min or more from surrounding eating occasions [18].

Data were analysed using GraphPad Prism v7.0d (GraphPad Software 2017, La Jolla CA, USA). The results of the analyses are presented as means and standard deviations (SD), with *p*-values of <0.05 on two-tailed tests being the threshold for statistical significance. Paired *t*-tests were used to test for differences in mean energy intake, macronutrient intake, and percentage of total daily energy and total daily macronutrient intake consumed during the nightshift in both the 2-Night Nurses Group and the combined All 2-Night Group. Variance in these parameters during the nightshift in the 3-Night Nurses Group was tested by two-way ANOVA, with nightshift and energy intake as factors.

## 3. Results

The study consisted of 20 nurses/midwives aged 30–54 years (mean ± SD, 42.7 ± 6.5 years), who completed 14-day dietary records and were included in the analysis. A summary of dietary intake is presented in Table 1.

### Energy Intake

**Figure 1:**
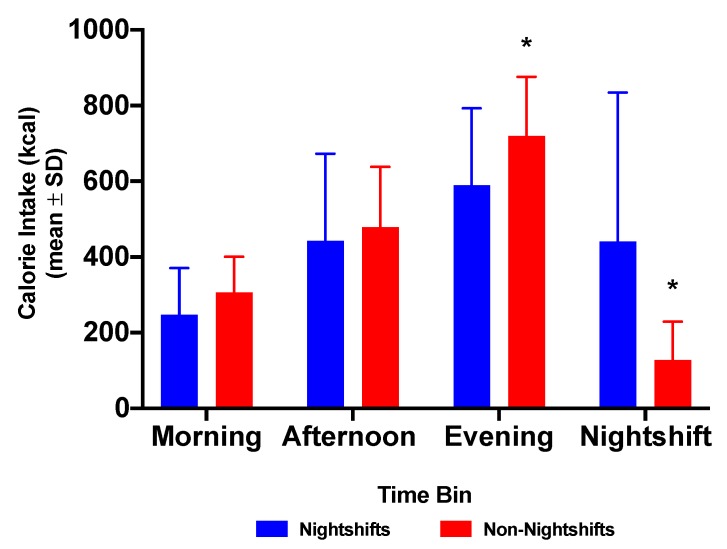
Energy intake (kcal) in each time bin between nightshifts (blue) and non-nightshifts (red) n = 20). Morning = 07:00–12:00 h; Afternoon = 12:00–17:00 h; Evening = 17:00–21:00 h; Nightshift = 21:00–07:00 h. * *p* < 0.05 for energy intake between 17:00–21:00 h and 21:00–07:00 h on non-nightshifts compared to the corresponding nightshift.

Group mean total daily energy intake (TDEI) was not significantly different between nightshifts and non-nightshifts (Table 1). Mean evening (17:00–21:00 h) energy intake in all nurses was not significantly different between nightshift and non-nightshifts (this trend *p* = 0.055). However, the proportion (%) of TDEI consumed during the evening period was significantly greater during the non-nightshifts than during nightshifts (Table 1). Mean energy intake during the nightshift was 466.5 ± 419.1 kcal, which was significantly greater (*p* < 0.001) than the mean energy intake (126.8 ± 102.0) for the same time period (21:00–07:00 h) during non-nightshifts (Figure 1, Table 1). 

**Figure 2:**
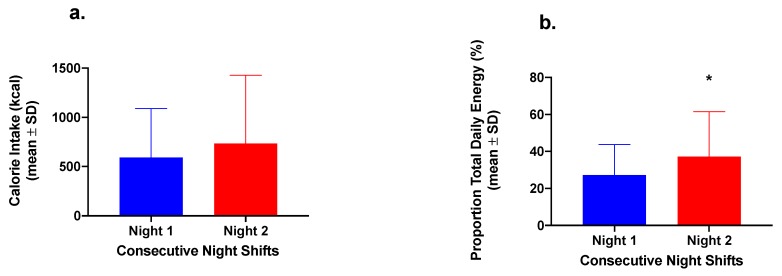
(**a**) Energy intake (kilocalories) and (**b**) proportion (as percentage) of total daily energy intake consumed during the nightshift phase (21:00–07:00 h) in nurses working two consecutive nightshifts only (n = 8). * *p* < 0.05 for the proportion of total daily energy compared to Night 1 (paired *t*-test).

In the 2-Night Nurses Group, there was no significant difference between Nights 1 and 2 in energy consumed during the nightshift (*p =* 0.276) (Figure 2). However, there was a significant difference in the proportion (%) of TDEI consumed during the nightshift (*p =* 0.037) in the 2-Night Nurses Group (Figure 2).

**Figure 3:**
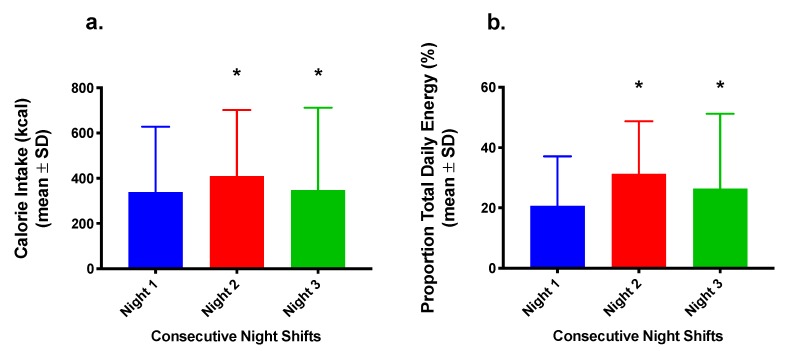
(**a**) Energy intake (kcal) and (**b**) proportion (as percentage) of total daily energy consumed during the nightshift phase (21:00–07:00 h) in nurses working three consecutive nightshifts (n = 12). * *p* < 0.05 for energy intake and proportion of total daily energy compared to Night 1 (two-way ANOVA).

The 3-Night Nurses Group showed a significant within-participant factor difference (*p* < 0.0001) in energy consumption between sequential night shifts (Figure 3). There was also a significant within-participant factor difference (*p =* 0.004) in the proportion of daily energy distributed to the nightshift (Figure 3).

**Figure 4:**
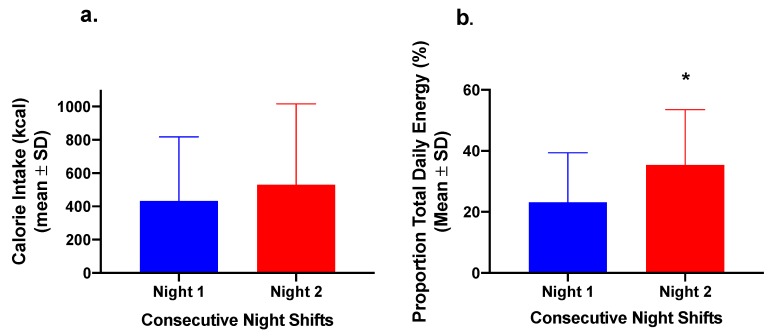
(**a**) Energy intake (kcal) and (**b**) proportion (as percentage) of total daily energy intake consumed during the nightshift phase (21:00–07:00 h) in the entire group of nurses together (n = 20). * *p* < 0.05 for the proportion of total daily energy compared to Night 1 (paired *t*-test).

To examine the trend of increasing intake between Nights 1 and 2 further, the nurses were analysed as a combined group (n = 20) to compare the difference in total energy and proportion of daily energy consumed over the first two nightshifts (Figure 4). While the difference in absolute energy intake did not differ significantly (*p =* 0.138), there was a significant difference in the proportion of total daily energy consumed during the nightshift between Nights 1 and 2 (*p =* 0.008) (Figure 4).

Changes in macronutrient distribution are also shown in Table 1. In the analysis of the combined group (All Group 2-Night), there was no overall significant difference in the intake of carbohydrate, fat, and protein, but evidence of a consistent trend toward increasing carbohydrate intake (Night 1: 212.8 ± 177.6 kcal; Night 2: 264.1 ± 228.6 kcal) and decreasing dietary fat intake (Night 1: 169.2 ± 160.0 kcal; Night 2: 157.6 ± 200.1 kcal) (Table 1). There were significant within-participant differences in the intakes of carbohydrate, fat, and protein across the nightshifts in the 3-Night group (Table 1). However, the respective contribution of carbohydrate, fat, and protein to energy consumed during the nightshift did not reach statistical significance, although there was a trend toward decreasing dietary fat across sequential nightshifts (Night 1: 118.8 ± 119.4 kcal; Night 2: 113.3 ± 112.9 kcal; Night 3: 74.5 ± 68.9 kcal) (Table 1). Protein made the least, and carbohydrate the greatest, contributions to energy intake during the nightshift in all groups (means 50.5 ± 11.8 kcal and 212 ± 51.3 kcal, respectively) (Table 1).

**Figure 5:**
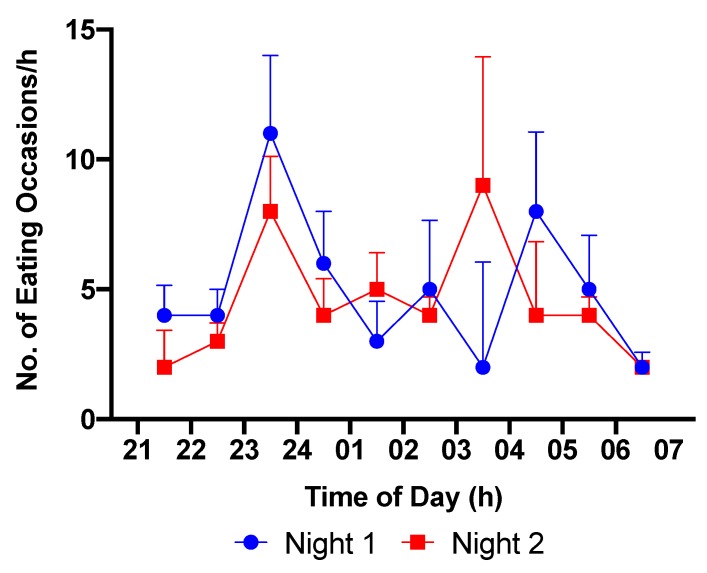
Eating occasions per hour during the nightshift phase (21:00–07:00 h) in the entire group of nurses together (n = 20).

Examination of the number and distribution of eating occasions across the nightshifts showed there to be no significant difference in the number of eating occasions during the nightshift between Nights 1 and 2. The distribution of eating occasions across Nights 1 and 2 is shown in Figure 5. On both nights there was a peak in the number of eating occasions between 23:00 and 24:00 h. A second peak was observed between 04:00 and 05:00, and 03:00 and 04:00 h on Nights 1 and 2, respectively.

## 4. Discussion

There was no difference in the intake of total daily energy between night and day shifts in the nurses on rotational shifts. However, the nurses redistributed their daily intake of energy into the nightshift period in increasing proportions, with a significant increase on Night 2 in the proportion of total daily energy consumed, as compared to Night 1.

The lack of difference in total daily energy intake is consistent with evidence for there being no difference in energy intake between day and nightshift workers from the wider literature [4,5,6,7]. Our study, however, showed a clear redistribution of energy, with energy intake in the entire group between 21:00 and 07:00 h increasing from 26% during the first nightshift to 33.5% on the second nightshift. In the nurses working three consecutive nights a similar increase was observed, from 21% on the first nightshift to 28.2% on the second, falling to 25.7% on the third night, which still exceeded the energy intake during the first night. These findings are in agreement with recent evidence to suggest that nurses working three consecutive nightshifts, redistributed their average intake of energy to the nightshift phase between 18:00 and 07:00 h [4]. In this study, the distribution of energy intake for night shift workers was significantly different between their days off and their working nights. In particular, while there was no significant peak of energy intake at any time period, the average intake of energy was redistributed into the evening and night phase between 18:00 and 07:00 h [4]. 

The redistribution of total daily energy to the nightshift period over sequential nights is relevant to the associations between increased cardiometabolic risk and nightshift work, independent of any observed increase in total energy intake. Of particular relevance is the fact that the metabolic effects of one meal are not independent of energy intake at previous or subsequent meals [19]. The consumption of low-calorie snacks after an earlier, larger meal during the nightshift may result in impaired postprandial lipaemia [19], and result in deleterious effects on LDL cholesterol [20]. In the present study, analysis of the patterns of macronutrient intake across sequential nights revealed a consistent increase in the intake of carbohydrate as a proportion of energy redistributed to the nightshift period.

In the present study, nurses consumed a greater proportion of energy in the evening (17:00–21:00 h) on non-nightshifts compared to nightshifts, though this did not reach statistical significance. In a study comparing fixed-day workers to shift workers working three consecutive nightshifts [4], both the fixed-day and shift workers consumed the greatest proportion of their daily energy between 18:00 and 21:00 h on their days off. However, on workdays, fixed day workers consumed their greatest proportion of energy between 12:00 and 15:00 h, while nightshift workers redistributed energy to the night-time (18:00–06:00 h) [4]. Our findings are in agreement with these previous observations. It is unlikely that circadian factors influence this pattern, with the evidence suggesting that the increased energy intake in the evening on non-nightshifts reflects a behavioural pattern. In the first instance, the fast rotating shift schedules covering 2–3 consecutive nightshifts worked by nurses are insufficient for circadian clock adaptation to the nightshift [21]. Secondly, on days off, both nightshift workers and fixed day workers have similar eating patterns [4], indicating that intermittent nightshifts do not dramatically alter behaviours of food intake on days off. While the pattern of energy intake during nightshifts is likely to primarily be behavioural, late meal timing can shift circadian rhythms in peripheral tissues such as the liver [22], which may influence metabolic responses when returning to day shifts or days off. Finally, the pattern of peak energy intake in the evening period is in accord with that of the UK population, which is characterised by increasing energy consumption over the course of the day with the greatest proportion of total daily energy (46%–48%) being consumed in the evening [23]. This is consistent with the mean total daily energy consumed by nurses (46%) in the evening of non-nightshifts in the present study [23].

Limitations of our study include the dietary recall records in shift workers being prone to both under and over-reporting [24]. The availability and ease of access to food on nightshifts and increasing awareness of health amongst nightshift workers [25] may have led to casual snacking during nightshifts going undocumented. The sample size of the study was small, in part because of the intensity of the study protocol, with daily self-assessment by the nurses and monitoring their families during work and nonwork periods. However, the sample size is comparable or larger than similar studies of shift workers and circadian misalignment protocols [4,26,27]. Finally, since the primary study was a mixed methods study encompassing both qualitative and quantitative methods, the latter of which focused on measures of sleep and stress, biomarkers of cardiometabolic were not measured.

## 5. Conclusions

In conclusion, our findings have demonstrated that during rotational nightshift work, energy intake is redistributed into the nightshift, while total daily energy remains unchanged. These data provide novel insight into the dynamic patterns of energy redistribution during consecutive nightshifts.

## Figures and Tables

**Table 1:** Energy Intake in Nurses Working 2-Night or 3-Night Consecutive Nightshifts Compared to Non-Nightshifts.

**Dietary Intake Nights vs. Non-Nights**	**Nightshifts**	**Non-Nightshifts**		***p* Value**
**Total daily energy, kcal**	1566.5 ± 557.2	1551.1 ± 327.4		0.919
**Morning energy 07:00–12:00 h, kcal**	246.8 ± 123.7	305.9 ± 94.7		0.053
**Morning energy, % TDEI**	15.8 ± 9.0	19.7 ± 4.6		0.034
**Afternoon energy 12:00–17:00 h, kcal**	443.4 ± 230.4	478.6 ± 159.4		0.538
**Afternoon energy, % TDEI**	28.3 ± 13.5	30.5 ± 6.9		0.844
**Evening energy 17:00–21:00 h, kcal**	588.7 ± 203.7	719.3 ± 156.9		0.055
**Evening energy, % TDEI**	38.8% ± 11.9%	46.4% ± 7.5%		0.044 *
**Nightshift energy 21:00–07:00 h, kcal**	466.5 ± 419.1	126.8 ± 102.0		0.001 *
**Nightshift energy 21:00–07:00 h, % TDEI**	29.8% ± 5.1%	8.2% ± 5.2%		0.0001 *
**2-Night Group Dietary Intake Over Nightshifts**	**Night 1**	**Night 2**		***p* value**
**2-Night Group (n = 8) energy, kcal**	591.5 ± 497.4	734.8 ± 692.0		0.276
**2-Night Group (n = 8) energy, %TDEI**	27.3% ± 16.5%	42.3% ± 18.2%		0.037 *
**All Group (n = 20) 2-Night energy, kcals**	440.5 ± 373.3	483.4 ± 487.0		0.138
**All Group (n = 20) 2-Night energy, %TDEI**	26.0% ± 15.7%	33.5% ± 20.2%		0.008 *
**All Group (n = 20) 2-Night CHO, kcal**	212.8 ± 177.6	264.1 ± 228.6		0.188
**All Group (n = 20) 2-Night CHO, %CHO energy**	25.4% ± 16.6%	38.1% ± 30.2%		0.070
**All Group (n = 20) 2-Night FAT, kcal**	169.2 ± 160.0	157.6 ± 200.1		0.686
**All Group (n = 20) 2-Night FAT, %FAT energy**	23.7% ± 17.2%	31.7% ± 29.3%		0.149
**All Group (n = 20) 2-Night PRO, kcal**	57.6 ± 51.1	65.4 ± 69.5		0.455
**All Group (n = 20) 2-Night PRO, %PRO energy**	20.5% ± 17.2%	28.9% ± 23.8%		0.126
**3-Night Group Dietary Intake Over Nightshifts**	**Night 1**	**Night 2**	**Night 3**	***p* value**
**3-Night Group (n = 12) energy, kcal**	326.0 ± 280.9	383.7 ± 294.6	338.7 ± 350.4	0.0001 **/0.549
**3-Night Group (n = 12) energy, %TDEI**	21.0% ± 16.1%	28.2% ± 18.3%	25.7% ± 23.8%	0.004 **/0.224
**3-Night Group (n = 12) CHO, kcal**	175.5 ± 148.2	226.2 ± 147.5	161.4 ± 127.6	0.001 **/0.236
**3-Night Group (n = 12) CHO, %CHO energy**	22.5% ± 16.4%	36.8% ± 34.6%	28.1% ± 28.0%	0.002 **/0.204
**3-Night Group (n = 12) FAT, kcal**	118.8 ± 119.4	113.3 ± 112.9	74.5 ± 68.9	0.014 **/0.380
**3-Night Group (n = 12) FAT, %FAT energy**	20.0% ± 17.4%	27.5% ± 31.0%	15.8% ± 15.0%	0.008 **/0.246
**3-Night Group (n = 12) PRO, kcal**	44.5 ± 39.3	50.3 ± 33.6	34.6 ± 30.3	0.024 **/0.390
**3-Night Group (n = 12) PRO, %PRO energy**	19.5% ± 19.2%	24.4% ± 14.3%	18.2% ± 19.6%	0.040 **/0.557

* Denotes statistical significance. ** Denotes *p*-value for within-participant significance. Kcal = kilocalories; TDEI = total daily energy intake; CHO = carbohydrates; FAT = dietary fat; PRO = protein; % = proportion of total daily energy as a percentage; 2-Night Group = nurses working only two consecutive nights; 3-Night Group = nurses working three consecutive nights; All Group 2-Night = all nurses analysed for differences between Night 1 and Night 2 (excluding Night 3 from the 3-Night Group).

## References

[1] Potter G., Skene D.J., Arendt J., Cade J., Grant P., Hardie L. (2016). Circadian rhythm and sleep disruption: Causes, metabolic consequences, and countermeasures. Endocr. Rev..

[2] Garaulet M., Ordovás J., Madrid J. (2010). The chronobiology, etiology and pathophysiology of obesity. Int. J. Obes..

[3] Depner C., Stothard E., Wright K. (2014). Metabolic consequences of sleep and circadian disorders. Curr. Diab. Rep..

[4] Molzof H., Wirth M., Burch J., Shivappa N., Hebert J., Johnson R., Gamble K. (2017). The impact of meal timing on cardiometabolic syndrome indicators in shift workers. Chronobiol. Int..

[5] Bonham M.P., Bonnell E.K., Huggins C.E. (2017). Energy intake of shift workers compared to fixed day workers: A systematic review and meta-analysis. Chronobiol. Int..

[6] Shaw E., Dorrian J., Coates A.M., Leung G.K.W., Davis R., Rosbotham E., Warnock R., Huggins C.E., Bonham M.P. (2019). Temporal pattern of eating in night shift workers. Chronobiol. Int..

[7] Esquirol Y., Bongard V., Mabile L., Jonnier B., Soulat J., Perret B. (2009). Shift work and metabolic syndrome: Respective impacts of job strain, physical activity, and dietary rhythms. Chronobiol. Int..

[8] Buysse D.J. (2014). Sleep health: Can we define it? Does it matter?. SLEEP.

[9] Johnston J.D. (2014). Physiological responses to food intake throughout the day. Nutr. Res. Rev..

[10] Bass J. (2012). Circadian topology of metabolism. Nature.

[11] Oike H., Oishi K., Kobori M. (2014). Nutrients, clock genes, and chrononutrition. Curr. Nutr. Rep..

[12] Young M., McGinnis G. (2016). Circadian regulation of metabolic homeostasis: Causes and consequences. Nat. Sci. Sleep.

[13] Westerterp-Plantenga M. (2016). Sleep, circadian rhythm and body weight: Parallel developments. Proc. Nutr. Soc..

[14] Lowson E., Middleton B., Arber S., Skene D.J. (2013). Effects of night work on sleep, cortisol and mood of female nurses, their husbands and children. Sleep Biol. Rhythm..

[15] Thompson E. (2009). Understanding How Night Work Influences the Everyday Family Lives of Nurses, Their Husbands and Children. Ph.D. Thesis.

[16] (2018). Nutritics, Research Edition (v5.09).

[17] Wrieden W., Barton K. Calculation and Collation of Typical Food Portion Sizes for Adults Aged 19-64 and Older People Aged 65 and Over. Final Technical Report to Food Standards Agency. http://www.foodbase.org.uk/results.php?f_category_id=&f_report_id=82.

[18] Leech R.M., Timperio A., Livingstone K.M., Worsley A., McNaughton S.A. (2017). Temporal eating patterns: Associations with nutrient intakes, diet quality, and measures of adiposity. Am. J. Clin. Nutr..

[19] Al-Naimi S., Hampton S., Richard P., Tzung C., Morgan L. (2004). Postprandial metabolic profiles following meals and snacks eaten during simulated night and day shift work. Chronobiol. Int..

[20] Hibi M., Masumoto A., Naito Y., Kiuchi K., Yoshimoto Y., Matsumoto M., Katashima M., Oka J., Ikemoto S. (2013). Nighttime snacking reduces whole body fat oxidation and increases LDL cholesterol in healthy young women. Am. J. Physiol. Regul. Integr. Comp. Physiol..

[21] Costa G., Ghirlanda G., Tarondi G., Minors D., Waterhouse J. (1994). Evaluation of a rapidly rotating shift system for tolerance of nurses to nightwork. Int. Arch. Occup. Environ. Health.

[22] Wehrens S., Christou S., Isherwood C., Middleton B., Gibbs M., Archer S., Skene D.J., Johnston J.D. (2017). Meal timing regulates the human circadian system. Curr. Biol..

[23] Almoosawi S., Vingeliene S., Karagounis L., Pot G. (2016). Chrono-nutrition: A review of current evidence from observational studies on global trends in time-of-day of energy intake and its association with obesity. Proc. Nutr. Soc..

[24] Chen Y., Lauren S., Chang B.P., Shechter A. (2019). Objective food intake in night and day shift workers: A laboratory study. Clocks Sleep.

[25] Bonnell E.K., Huggins C.E., Huggins C.T., McCaffrey T.A., Palermo C., Bonham M.P. (2017). Influences on dietary choices during day versus night shift in shift workers: A mixed methods study. Nutrients.

[26] Morris C., Purvis T., Mistretta J., Scheer F. (2015). Effects of the internal circadian system and circadian misalignment on glucose tolerance in chronic shift workers. J. Clin. Endocrinol. Metab..

[27] Scheer F., Hilton M., Mantzoros C., Shea S. (2009). Adverse metabolic and cardiovascular consequences of circadian misalignment. Proc. Natl. Acad. Sci. USA.

